# Do BARD1 Mutations Confer an Elevated Risk of Prostate Cancer?

**DOI:** 10.3390/cancers13215464

**Published:** 2021-10-30

**Authors:** Klaudia Stempa, Dominika Wokołorczyk, Wojciech Kluźniak, Emilia Rogoża-Janiszewska, Karolina Malińska, Helena Rudnicka, Tomasz Huzarski, Jacek Gronwald, Katarzyna Gliniewicz, Tadeusz Dębniak, Anna Jakubowska, Marcin Lener, Joanna Tomiczek-Szwiec, Paweł Domagała, Malwina Suszynska, Piotr Kozlowski, Tomasz Kluz, Mariusz Naczk, Jan Lubiński, Steven A. Narod, Mohammad R. Akbari, Cezary Cybulski, on behalf of the Polish Hereditary Prostate Cancer Consortium

**Affiliations:** 1 International Hereditary Cancer Center, Department of Genetics and Pathology, Pomeranian Medical University in Szczecin, 71-252 Szczecin, Poland; dominikawok@o2.pl (D.W.); kluzniak@pum.edu.pl (W.K.); emilia.rogoza.janiszewska@pum.edu.pl (E.R.-J.); karolina.malinska@pum.edu.pl (K.M.); helena.rudnicka@pum.edu.pl (H.R.); huzarski@pum.edu.pl (T.H.); jgron@pum.edu.pl (J.G.); katarzynagliniewicz@gmail.com (K.G.); debniak@pum.edu.pl (T.D.); aniaj@pum.edu.pl (A.J.); marcinlener@poczta.onet.pl (M.L.); lubinski@pum.edu.pl (J.L.); 2 Department of Clinical Genetics and Pathology, University of Zielona Góra, 65-417 Zielona Góra, Poland; 3 Independent Laboratory of Molecular Biology and Genetic Diagnostics, Pomeranian Medical University in Szczecin, 71-252 Szczecin, Poland; 4 Department of Histology, Department of Biology and Genetics, Faculty of Medicine, University of Opole, 45-040 Opole, Poland; jszwiec@uni.opole.pl; 5 Department of Pathology, Pomeranian Medical University in Szczecin, 71-252 Szczecin, Poland; pawel.domagala@pum.edu.pl; 6 Department of Molecular Genetics, Institute of Bioorganic Chemistry, Polish Academy of Sciences, 61-704 Poznan, Poland; msuszynska@man.poznan.pl (M.S.); kozlowp@ibch.poznan.pl (P.K.); 7 Department of Gynecology and Obstetrics, Institute of Medical, Sciences, Medical College of Rzeszow University, 35-959 Rzeszow, Poland; jtkluz@interia.pl; 8 Collegium Medicum, University of Zielona Gora, 65-417 Zielona Gora, Poland; m.naczk@cm.uz.zgora.pl; 9 Women’s College Research Institute, Women’s College Hospital, University of Toronto, Toronto, ON M5G 1N8, Canada; steven.narod@wchospital.ca (S.A.N.); mohammad.akbari@utoronto.ca (M.R.A.); 10 Dalla Lana School of Public Health, University of Toronto, Toronto, ON M5S 1A1, Canada

**Keywords:** *BARD1*, mutation, prostate cancer, risk, survival

## Abstract

**Simple Summary:**

Current cancer testing gene panels tend to be comprehensive. One of the genes commonly included in the testing panels is *BARD1*. To establish whether *BARD1* mutations predispose to prostate cancer, we sequenced *BARD1* in 390 hereditary prostate cancer cases, genotyped 5715 men with unselected prostate cancer and 10,252 controls for three recurrent rare *BARD1* variants in Poland. We did not see an elevated prostate risk cancer given p.Q564X truncating mutation, p.R658C missense mutation and p.R659= synonymous variant. Neither variant influenced prostate cancer characteristics or survival. Our study is the first to evaluate the association between *BARD1* mutations and prostate cancer susceptibility. It is not justified to inform men about increased prostate cancer risk in case of identification of a *BARD1* mutation. However, a female relative of a man with a *BARD1* mutation may benefit from this information and be tested, because *BARD1* is a breast cancer susceptibility gene.

**Abstract:**

The current cancer testing gene panels tend to be comprehensive rather than site-specific. *BARD1* is one of the genes commonly included in the multi-cancer testing panels. Mutations in *BARD1* confer an increase in the risk for breast cancer, but it is not studied whether or not they predispose to prostate cancer. To establish if *BARD1* mutations also predispose to prostate cancer, we screened *BARD1* in 390 Polish patients with hereditary prostate cancer. No truncating mutations were identified by sequencing. We also genotyped 5715 men with unselected prostate cancer, and 10,252 controls for three recurrent *BARD1* variants, including p.Q564X, p.R658C and p.R659=. Neither variant conferred elevated risk of prostate cancer (ORs between 0.84 and 1.15, *p*-values between 0.57 and 0.93) nor did they influence prostate cancer characteristics or survival. We conclude that men with a *BARD1* mutation are not at elevated prostate cancer risk. It is not justified to inform men about increased prostate cancer risk in case of identification of a *BARD1* mutation. However, a female relative of a man with a *BARD1* mutation may benefit from this information and be tested for the mutation, because *BARD1* is a breast cancer susceptibility gene.

## 1. Introduction

Prostate cancer is the most heritable cancer in men (estimated heritability 57%) [1,2,3]. The high heritability is attributable to the additive effects of multiple low-risk single nucleotide polymorphisms (SNPs) and to rare germline mutations in a number of susceptibility genes [4,5,6,7,8,9,10]. Therefore, testing for elevated risk of prostate cancer can be conducted based on SNPs personalized risks scores (PRS) and cancer gene panels. 

Recent multiancestry meta-analysis of prostate cancer genome-wide association studies including 107,247 cases and 127,006 controls identified 86 new genetic risk variants independently associated with prostate cancer risk, bringing the total to 269 known risk variants. The top genetic risk score decile was associated with odds ratios that ranged from 5.06 (95% confidence interval (CI) 4.84–5.29) for men of European ancestry to 3.74 (95% CI 3.36–4.17) for men of African ancestry. This study supported the PRS as an important approach for personalized risk prediction [11].

Several prostate cancer susceptibility genes have been identified to date, including those associated with high risk of prostate cancer (*BRCA2* and *HOXB13*-up to eight-fold overall risk), moderate risk (*BRCA1*-up to 3.8-fold; DNA mismatch repair genes *MLH1*, *MSH2*, *MSH6*, *PMS2* and *EPCAM* up to 2.3 fold increased for all males from Lynch syndrome families), and the genes with limited data regarding risk estimates including *CHEK2*, *ATM* and *NBN*. There are also genes which were associated only with aggressive disease (i.e., *PALB2*) or genes which mutations were detected only in metastatic prostate cancer (i.e., *RAD51C-D*, *BRIP1*, Fanconi anemia genes). It should be considered that the spectrum of mutations in prostate cancer susceptibility genes may be different in diverse populations. There are race-associated germline mutations/variants, i.e., *RET* and *SMAD4* mutations may be unique to African-American men [12]. Germline mutation testing becomes important for prostate cancer treatment, management and hereditary cancer assessment. Testing panels include genes with strong, limited, and unknown risk for prostate cancer and also genes conferring risks for other cancer types. Recently, recommendations for panel choice and priority genes to test in men with prostate cancer and men at high risk for prostate cancer were developed [12]. However, the current multigene genetic testing options include focused, guideline-based, comprehensive, and reflex panels, and it is challenging to make a clear decision which panel to choose depending on particular clinical situation (i.e., for men with metastatic disease, nonmetastatic disease, men at increased prostate cancer risk) to balance the benefits and limitations of expanded testing [12]. In particular, this issue applies to comprehensive panels which analyze many genes associated with multisite cancer susceptibility. For example, The Invitae Common Hereditary Cancers panel tests for mutations in forty-seven genes, which are associated with, e.g., hereditary prostate cancer, hereditary breast and/or ovarian cancer, and Lynch syndrome. 

One of the genes commonly included in the testing panels is *BARD1* (*BRCA1* associated RING domain 1 gene). Mutations in the *BARD1* gene confer a two- to three-fold elevated breast cancer risk, but it has not been determined whether or not they predispose to prostate cancer [13]. This is important for male members of families where a testing panel has revealed a *BARD1* mutation and for genetic counselors who consider offering testing to male family members. In the event that a *BARD1* mutation is found, the men will wish to know if they face an elevated risk for prostate cancer.

BARD1 acts in the *BRCA1* gene dependent pathway by forming heterodimers with BRCA1. The interaction between the two proteins is essential for maintaining the stabilization of the BRCA1 protein as well as for the newly formed BRCA1-BARD1 complex. [14]. This complex has activity of E3 ubiquitin ligase, is involved in DNA damage repair, cell cycle control, regulation of hormone signaling, and apoptosis [15]. Because *BRCA1* is a prostate cancer susceptibility gene (with an estimated risk for prostate cancer of two- to four-fold by the age of 65) [16,17,18,19], and the BRCA1 protein interacts with the BARD1 protein, we considered *BARD1* to be a candidate prostate cancer susceptibility gene. We sequenced the *BARD1* gene in 390 Polish men with hereditary prostate cancer. Then, we genotyped 5715 patients with unselected prostate cancer, and 10,252 cancer-free controls for three recurrent variants of *BARD1* in the Polish population including one truncating variant (c.1690C>T; p.Q564X), one missense mutation (c.1972C>T; p.R658C), and one synonymous substitution (c.1977A>G; p.R659=). 

## 2. Results

First, we fully sequenced the *BARD1* gene in 390 Polish patients from families with hereditary prostate cancer. No truncating mutations were identified.

Then, we genotyped 5715 patients with prostate cancer and 10,252 cancer-free controls for three rare changes in *BARD1*, (i) one definitive truncating mutation (c.1690C>T; p.Q564X) with proved effect on breast cancer risk, and two variants of unknown significance (ii) a missense substitution (c.1972C>T; p.R658C) and (iii) one synonymous substitution (c.1977A>G; p.R659=). A truncating *BARD1* mutation p.Q564X was detected in 7 (0.12%) men with prostate cancer compared to 15 (0.15%) cancer-free individuals (OR = 0.84, *p* = 0.87). This mutation was not detected in any of the 711 familial prostate cancer cases (from the 5715 unselected prostate cancer cases) (*p* = 0.62) (Table 1). Then, we analyzed clinical characteristics of patients with prostate cancer who carried *BARD1* p.Q564X truncating mutation to those in non-carriers. We saw no difference in the age of diagnosis in mutation carriers compared to non-carriers (mean age of onset was 68.9 versus 67.3, respectively; *p* = 0.61). Additionally, PSA at the time of diagnosis, Gleason score and tumor stage were similar in patients with the p.Q564X variant compared to non-carriers (Table 2).

Neither of the other two variants of the *BARD1* gene, a missense substitution p.R658C and a synonymous change p.R659=, were associated with elevated prostate cancer risk. 

A missense variant p.R658C of *BARD1* was detected in 39 (0.68%) unselected prostate cancer cases compared to 61 (0.60%) cancer-free controls (OR = 1.15, *p* = 0.57). The variant was present in 3 (0.42%) of 711 men with familial prostate cancer (from unselected cancers) (OR = 0.71, *p* = 0.74) (Table 1).

We saw no difference in the age of diagnosis in p.R658C variant carriers compared to non-carriers (mean age of onset was 68.1 versus 67.3, respectively; *p* = 0.52). In addition, PSA at the time of diagnosis, Gleason score and tumor stage were similar in patients with the p.R658C missense mutation compared to non-carriers (Table 2).

A synonymous variant p.R659= of *BARD1* was detected in 19 (0.33%) men with unselected prostate cancer and in 35 (0.34%) cancer-free controls (OR = 0.97, *p* = 0.93). It was identified in 0.56% of 711 familial prostate cancer cases, but the difference was marginal (OR = 1.65), and not statistically significant (*p* = 0.53) (Table 1). The characteristics of patients affected by prostate cancer with and without the p.R659= variant was similar. The mean age of onset in patients with *BARD1* p.R659= variant versus that seen in non-carriers was similar (64.6 versus 67.3, respectively; *p* = 0.18). The PSA level at the time of diagnosis, Gleason score and tumor stage were also similar in men with the p.R659= variant compared to non-carriers.

**Figure 1:**
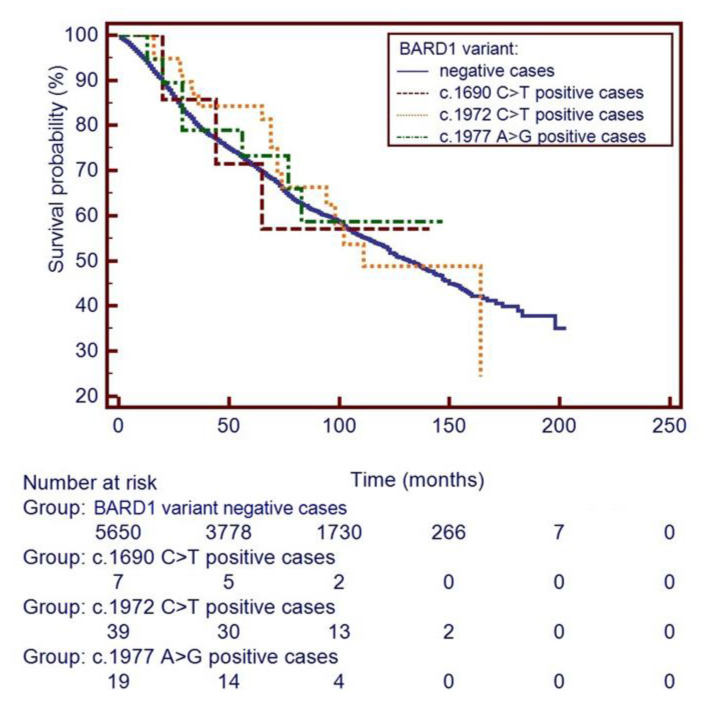
Survival curves of men with prostate cancer and a *BARD1* variant (7 patients with c.1690C>T, 39 patients with c.1972C>T and 19 men with c.1977A>G) and of *BARD1* variant negative prostate cancer patients (5650 non-carriers). Footnote to Figure 1: *BARD1* variant negative prostate cancer patients –prostate cancer patients negative for c.1690 C>T, c.1972 C>T and c.1977 A>G variants of *BARD1*.

During the follow up time of 102 months, there were 3 deaths recorded in the 7 *BARD1* p. Q564X mutation carriers (42.9%; *p* = 0.98), 17 deaths in 39 (43.6%, *p* = 0.88) missense variant p.R658C carriers, 7 deaths in 19 (36.8%, *p* = 0.79) p.R659= variant carriers, compared to 2397 deaths among the 5650 non-carriers (42.4%), but neither difference was significant. Kaplan–Maier curves for patients with each of 3 *BARD1* variants and for non-carriers are presented in Figure 1.

We did not see a statistical difference in 5-year survival for carriers of three *BARD1* variants-p.Q564X, p.R658C and p.R659= (survival 71%, 83% and 73%, respectively) compared to non-carriers (72%), all differences were not significant (*p*-values between 0.11 and 0.97). Additionally, 10-year survival for carriers of the three *BARD1* variants (survival 57%, 55% and 49%, respectively) was similar to that seen in non-carriers (54%), (all *p*-values between 0.82 and 0.98). The unadjusted and adjusted for age hazard ratios (HRs) for death associated with each of the three *BARD1* variants are provided in Table 3. Crude HRs for the p.Q564X, p.R658C and p.R659= variants were 0.95, 0.94 and 0.92, respectively, and neither was significant.

**Figure 2:**
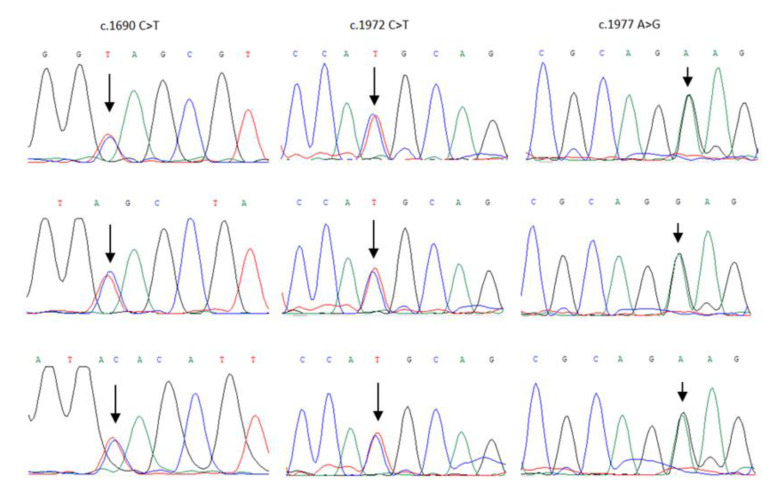
LOH analysis in tumors from nine patients with *BARD1* variants (c.1690 C>T, c.1972 C>T and c.1977 A>G). LOH was not observed in any of the nine studied prostate cancers. Localization of *BARD1* variants is marked with an arrow (↓).

We were able to analyze tumor DNA of three men with *BARD1* truncating mutation p.Q564X, three patients with a missense variant p.R658C, and three men with a synonymous change p.R659= for LOH at the *BARD1* locus. Sanger sequencing did not show LOH in any tumor of 9 analyzed patients (Figure 2).

## 3. Discussion

The *BARD1* gene is located on chromosome 2 (locus 2q34-35). It consists of 11 exons. The gene encodes a 777-amino acid protein. The BARD1 protein contains N-terminal RING domain with a zinc finger motif, three ankyrin repeat domains (ANK) in the central part of the protein, and two BRCT domains at the C-terminal end. The BARD1 and BRCA1 proteins form a heterodimer complex. The interaction between the two proteins is essential for maintaining the stabilization of BRCA1 [14,19,20,21]. The BRCA1/BARD1 heterodimer acts in the DNA damage response signaling pathway, cell cycle control, hormone signaling modulation and chromatin structural maintenance [22,23]. For example, BRCA1 and BARD1 bind DNA and interact with RAD51, BRCA1–BARD1 complex enhances the recombinase activity of RAD51, and have a role in homologous recombination repair [24]. BARD1 also plays a role in the maintenance of genome integrity by interacting with other proteins including p53. [25,26]. 

To date, little is known about cancer susceptibility in carriers of *BARD1* mutations. The most commonly studied is the association between *BARD1* and breast cancer [19,20,27]. Most recent, large association analysis of approximately 110,000 women performed by Breast Cancer Association Consortium provided evidence of an association between truncating *BARD1* mutation and an elevated risk of breast cancer [13]. In that study, the authors identified 62 carriers of truncating *BARD1* mutations in 58,728 women with breast cancer compared to 32 carriers in 52,976 controls (OR = 2.09; 95% CI, 1.35 to 3.23; *p* = 0.0001). Common variants in *BARD1* have also been related to a genetic susceptibility to neuroblastomas, colorectal and lung cancer but based on single reports [28,29,30]. Therefore, the evidence of the association of *BARD1* mutations with a genetic predisposition to cancers other than breast cancer is controversial. There are no studies to date which reported the influence of *BARD1* mutations on prostate cancer risk.

We selected three Polish *BARD1* rare recurrent variants for large case–control study: a founder deleterious mutation p.Q564X, localized in exon 8 of the *BARD1* gene, and two single base substitutions of unknown function, a missense mutation p.R658C and a synonymous change p.R659=, both located in exon 10 of *BARD1*, previously detected in our population [20]. ClinVar defines the p.Q564X mutation as clearly pathogenic. There are conflicting predictions regarding the clinical significance of *BARD1* p.R658C and p.R659R changes. ClinVar reports these two variants (p.R658C, p.R659=) as of unknown significance, likely benign or even benign. However, both variants are localized in an important domain of the gene (BRCT domain), and some studies refer to p.R658C and p.R659= as potentially pathological [31,32,33], so we choose to analyze prostate cancer risk given these two variants as well.

In the current study, we did not observe an increased risk for prostate cancer given any of the three *BARD1* variants studied. We also showed that these variants (including a truncating *BARD1* mutation) did not influence prostate cancer characteristics or survival among the carriers. Finally, we did not observe LOH at the *BARD1* locus in prostate cancers. In aggregate, our study suggests that *BARD1* mutations do not predispose to prostate cancer.

Most interestingly, we found no association of the Polish founder *BARD1* deleterious mutation (p.Q564X) with elevated risk for prostate cancer (OR = 0.84, 95%CI 0.34–2.05). Previously, we reported that the p.Q564X mutation conferred a two-fold increased risk of breast cancer (*p* = 0.04) [20]. In the current study, we also analyzed the frequency of *BARD1* variants in prostate cancer patients with relatives with breast cancer. There were 407 men with prostate cancer and a positive history of breast cancer in first or second degree relatives. Among these men, the p.Q564X mutation frequency was significantly higher than that seen in 10,252 cancer-free controls (0.74% vs. 0.15%; OR = 5.07, *p* = 0.026). In contrast, the p.R658C missense variant frequency was similar in prostate cancer patients with a family history of breast cancer and in cancer-free controls (0.49% vs. 0.60%; OR = 0.83, *p* = 0.79). The prevalence of the p.R659= variant was 0% in prostate cancer patients with a family history of breast cancer compared to 0.34% in cancer-free controls (OR = 0.35, *p* = 0.46). These results suggest that the p.Q564X deleterious mutation (but not p.R658C and p.R659= variants) predisposes to breast cancer, however this truncating mutation does not increase prostate cancer risk.

Our results that *BARD1* mutations do not confer increased risk of prostate cancer are in line with several previous reports. No mutations were detected in *BARD1* by Invitae by sequencing in 140 African-American patients with prostate cancer and 1695 Caucasian American men with prostate cancer [34]. In a study from the UK of 1281 early-onset cases and 1160 selected controls, no pathogenic for prostate cancer *BARD1* mutations were reported [35]. In a large study of 5545 European-ancestry men with prostate cancer, only nine (0.16%) patients carried a *BARD1* mutation (six different *BARD1* mutations) [36]. No *BARD1* mutations were detected in 692 men with metastatic prostate cancer unselected for age and family history of cancer [37]. In another study of 704 men with metastatic prostate cancer, only one *BARD1* mutation was detected (0.14%) compared to 0.09% in 54,802 reference controls from Genome Aggregation database (*p* = 0.46) [38].

The Polish population is genetically homogenous because it is populated by ethnic Poles. *BARD1* p.Q564X founder truncating mutation is seen in 0.15% of the general population of Poland. The presence of this deleterious founder allele enabled us to analyze its association with the risk of prostate cancer in a population based study at reasonable costs. In other populations, founder mutations in *BARD1* were not detected and truncating mutations of this gene are very uncommon, identified with an aggregate frequency of about 0.05% or less (different rare truncating mutations combined) [13,19]. Therefore, analysis of the association between *BARD1* truncating mutations and prostate cancer risk in other populations would require sequencing of the entire gene in large case–control study (given the ≤0.05% mutation frequency in other ethnic groups, at least approximately 15,000 cases and 30,000 controls should be screened, to have comparable power to our current study).

The imitations of our study include the low number of mutation carriers, so the results of the association of *BARD1* variants with clinical characteristics and survival should be interpreted with caution. Additionally, our control group was suboptimal. The aim of the control group was to estimate the frequency of *BARD1* variants in the underlying Polish population. We used both males and females to maximize the number of controls, because the studied variants are rare in the population. Our cases were older than controls, and our controls included both cancer-free men and women. However, both cases and controls were ethnic Poles, and the variants frequency (for each *BARD1* variant) was not dependent on age or sex. Therefore, we believe that our population controls are good approximation of age- and sex-matched cancer-free control group.

## 4. Materials and Methods

### 4.1. Patients

We studied two non-overlapping series of men with prostate cancer: 390 men with familial prostate cancer and 5715 men with unselected prostate cancer. The first series of cases was derived from a registry of familial cases housed at the Hereditary Cancer Center in Szczecin in Poland. We selected families with strong aggregations of prostate cancer, and those with youngest age of onset in probands and their relatives. The mean number of prostate cancers per family was 2.8. The mean age of diagnosis among the 390 men was 61.6 years (range 38–82). The series is described in detail previously [39].

The second series of cases included 5715 men with prostate cancer who were diagnosed between 1999 and 2015 in 14 centers situated throughout Poland. This study was initiated in Szczecin in 1999 and was extended to include Białystok and Olsztyn in 2002 and Opole in 2003. Other centers began recruiting between 2005 and 2008 (Koszalin, Gdansk, Lublin, Łodź, Warszawa, Wrocław, Poznan, Rzeszów, Bydgoszcz, Zabrze). Men with newly diagnosed treatment-naive prostate cancer were invited to participate. Prostate cancer cases enrolled in the study are unselected for age, clinical characteristics (stage, grade, PSA level at time of diagnosis), family history and treatment. Study subjects were asked to participate at the time of diagnosis. The patient participation rate was 85.4%. Patients who provided a blood sample within 6 months of diagnosis were enrolled. Men who provided blood sample above 6 months from diagnosis (*n* = 63) were excluded. The mean age of diagnosis was 67.3 years (range 35–96 years). A family history was taken either by the construction of a family tree or the completion of a standardized questionnaire. All first- and second-degree relatives diagnosed with prostate cancer and the ages of diagnosis were recorded. A total of 711 men reported at least one first- or second-degree relative with prostate cancer (familial cases). In addition, information was obtained on PSA level at time of diagnosis, grade (Gleason score) and stage, when possible. The vital status (dead or alive) and the date of death of the cases were requested from the Polish Ministry of the Interior and Administration in June 2016 and were obtained in July 2016. Survival data were obtained for all men with prostate cancer.

### 4.2. Controls

The control group included 10,252 cancer-free adults from the genetically homogeneous population of Poland. The control group consisted of 5545 cancer-free men (age 19–97 years, mean 59.0 years; 1486 men below age 50 years and 4059 men at age 50 or above) and 4707 cancer-free women (age 18 to 94 years, mean 54.0 years; 1865 women below age of 50 years and 2842 women at age 50 or above) from the International Hereditary Cancer Center (IHCC) cohort (Szczecin, Poland) [40,41,42,43]. The aim of the control group was to estimate the frequency of the three *BARD1* variants in the Polish population. The variants frequencies in controls were not dependent on age or sex. All cases and controls were ethnic Poles. The study was approved by the Ethics Committee of the Pomeranian Medical University in Szczecin. 

### 4.3. Exome Sequencing

The first series of 390 men with hereditary prostate cancer were tested by exome sequencing as described previously [39]. In brief, The Agilent SureSelect human exome kit (V6) was used for capturing sequence target regions. Paired-end sequencing was performed on a high throughput sequencing cartridge of Illumina NextSeq 500. The mean depth of coverage was approximately 100× (range 62× to 171×). On average, 98.8% (range 87.6% to 99.0%) of the CCDS exons were covered at 20x depth of coverage and higher which used for variant calling. We sought to identify protein truncating variants, variants at the consensus splice site likely to be dysfunctional and known pathogenic missense or intronic mutations in *BARD1* gene based on the literature, ClinVar and Human Genome Mutation Database.

### 4.4. Genotyping

5715 men with unselected prostate cancer and 10,252 controls were genotyped for the three variants of *BARD1* (c.1690 C>T, p.Q564X; c.1972 C>T, p.R658C; c.1977 A>G, p.R659=). Genomic DNA was isolated from 5 to 10 mL of peripheral blood. *BARD1* variants were genotyped using TaqMan assay (Thermo Fisher Scientific, Waltham, MA) using LightCycler^®^ Real-Time PCR 480 System (Roche Life Science, Indianapolis, IN). Laboratory technicians were blinded to case–control status. The primer and probe sequenced used in TaqMan-PCR assays are published in our previous report describing the association between *BARD1* variants and breast cancer [20]. TaqMan assays for the c.1690 C>T (p.Q564X) mutation were previously validated by an analysis of 18 randomly selected mutation carriers and 20 non-carriers by Sanger sequencing analysis on an ABI Prism 3130 genetic analyzer (Applied Biosystems, Carlsbad, CA, USA) according to the manufacturer’s protocol. The c.1972 C>T (p.R658C) and c.1977 A>G (p.R659=) variants were validated by the sequencing of 10 carriers and 10 non-carriers (for each variant). The results of TaqMan-PCR genotyping and direct sequencing validation were fully concordant [20].

### 4.5. Statistical Analysis

The prevalence of the *BARD1* alleles was estimated in prostate cancer cases and population controls and compared. Odds ratios were generated from two-by-two tables and statistical significance was assessed using the Fisher exact test or the chi-squared test where appropriate. Men with prostate cancer, with and without a *BARD1* variant were compared for age at diagnosis, family history and clinical features of the prostate cancers. Means were compared using t-test, and medians were compared using Mann–Whitney test. To estimate survival, Kaplan–Meier curves were constructed. Men were followed from the date of diagnosis (the date of biopsy) until the date of death or July 2016. The median follow up time was 102 months. The vital status was available for all men with prostate cancer. The crude hazard ratio for death (all-cause mortality), for each of three *BARD1* variants was estimated by means of the Cox proportional hazards model. Then, the data was re-analyzed, adjusting for age of diagnosis while also using the Cox proportional hazards model.

### 4.6. Loss of Heterozygosity Analysis

Formalin-fixed paraffin-embedded (FFPE) tissue samples were analyzed for nine carriers of *BARD1* variants, three of a truncating mutation c.1690C>T (p.Q564X), three of a missense variant c.1972C>T (p.R658C) and three of a synonymous variant c.1977A>G (p.R659=). FFPE tissue samples were obtained from Centers in Szczecin, Opole and Rzeszów. A pathology review of the samples was conducted by a pathologist associated with the study. Loss of heterozygosity (LOH) analysis at the *BARD1* locus was performed in DNA isolated from micro-dissected tumors from nine patients using the methodology described previously [7] with some modifications: (1) DNA was isolated with QIAamp DNA FFPE Tissue Kit (from QIAGEN); (2) For the c.1690 C>G mutation, LOH was analyzed by Sanger sequencing of 190 bp DNA fragment containing the variant using forward primer 5′ TGTCTTTGTCTAGTCGTCTAATGTTT and reverse primer 5′ GCCCACTGCCTATAAGTACAAGA); for the c.1972 C>T and c.1977 A>G variants LOH was analyzed by Sanger sequencing of 166 bp fragment containing both variants using forward primer 5′ GTGCTCACTTGATACTTAGTTTGC and reverse primer 5′GTTGTATTAAAAGAAAAATACCAGCTG.

## 5. Conclusions

This study is the first to evaluate the role of *BARD1* in genetic susceptibility to prostate cancer. It is not justified to inform men about increased prostate cancer risk in case of identification of a *BARD1* mutation using currently existing comprehensive gene testing panels. However, a female relative of a man with a *BARD1* mutation may benefit from this information and she may be tested for this mutation, because *BARD1* is a breast cancer susceptibility gene.

## Figures and Tables

**Table 1:** Prevalence of *BARD1* variants in 5715 patients with prostate cancer and 10,252 cancer-free controls.

Group	Number Total	Number Positive	%	OR	95% CI	*p*-Value
**Unselected Cases**						
c.1690 C>T	5715	7	0.12%	0.84	0.34–2.05	0.87
c.1972 C>T	5715	39	0.68%	1.15	0.77–1.72	0.57
c.1977 A>G	5715	19	0.33%	0.97	0.56–1.70	0.93
Familial cases						
c.1690 C>T	711	0	0.00%	0.46	0.03–7.78	0.62
c.1972 C>T	711	3	0.42%	0.71	0.22–2.26	0.74
c.1977 A>G	711	4	0.56%	1.65	0.59–4.66	0.53
Controls						
Males						
c.1690 C>T	5545	8	0.14%			
c.1972 C>T	5545	35	0.63%			
c.1977 A>G	5545	21	0.38%			
Females						
c.1690 C>T	4707	7	0.15%			
c.1972 C>T	4707	26	0.55%			
c.1977 A>G	4707	14	0.30%			
All controls						
c.1690 C>T	15	10,252	0.15%	Ref.	-	-
c.1972 C>T	61	10,252	0.60%	Ref.	-	-
c.1977 A>G	35	10,252	0.34%	Ref.	-	-

Familial cases (*n* = 711) are derived from a series of unselected cases.

**Table 2:** Characteristics of patients with prostate cancer who carry a *BARD1* variant (7 patients with c.1690C>T, 39 patients with c.1972C>T and 19 men with c.1977A>G) compared to *BARD1* variant-negative prostate cancer cases (5650 non-carriers).

Category		c.1690 C>T Positive Cases (*n* = 7)	*p*-Value	c.1972 C>T Positive Cases (*n* = 39)	*p*-Value	c.1977 A>G Positive Cases (*n* = 19)	*p*-Value	BARD1 * Variant Negative Cases (*n* = 5650)
Age of diagnosis	mean (range)	68.9 (64–77)	0.61	68.1 (51–93)	0.52	64.6 (47–77)	0.18	67.3 (35–93)
	<60	0.0% (0/7)	0.60	12.8% (5/39)	0.50	15.8% (3/19)	0.99	18.5% (1047/5650)
	61–70	71.4% (5/7)	0.29	51.3% (20/39)	0.48	68.4% (13/19)	0.06	44.4% (2510/5650)
	>70	28.6% (2/7)	0.94	35.9% (14/39)	0.88	15.8% (3/19)	0.09	37.0% (2093/5650)
PSA at diagnosis	median (range)	12.0 (3.1–443.3)	0.66	11.0 (0.24–104.0)	0.22	10.7 (0.33–42.0)	0.93	10.7 (0.1–5000)
	≤4.0	14.3% (1/7)	0.89	2.7% (1/37)	0.64	11.1% (2/18)	0.66	5.9% (153/2614)
	4.1–10	28.6% (2/7)	0.75	37.8% (14/37)	0.76	38.9% (7/18)	0.81	41.6% (1089/2614)
	10.1–20.0	28.6% (2/7)	0.81	24.3% (9/37)	0.96	27.8% (5/18)	0.97	24.6% (644/2614)
	>20.0	28.6% (2/7)	0.97	35.1% (13/37)	0.43	22.2% (4/18)	0.79	27.9% (728/2614)
Gleason score	<7	42.9% (3/7)	0.82	50.0% (18/36)	0.72	41.2% (7/17)	0.40	54.4% (1647/3028)
	7	28.6% (2/7)	0.65	27.8% (10/36)	0.48	41.2% (7/17)	0.09	27.4% (830/3028)
	>7	28.6% (2/7)	0.82	22.2% (8/36)	0.68	17.6% (3/17)	0.95	18.2% (551/3028)
Stage	T1/2	71.4% (5/7)	0.80	78.8% (26/33)	0.83	80.0% (12/15)	0.92	75.6% (1726/2283)
	T3/4	28.6% (2/7)	0.79	21.2% (7/33)	0.83	20.0% (3/15)	0.92	24.4% (557/2283)
Positive family history	positive	0.0% (0/7)	0.55	10.0% (3/30)	0.59	23.5% (4/17)	0.54	15.2% (704/4633)

*—Reference group for *p*-value calculations.

**Table 3:** Survival of prostate cancer patients with a *BARD1* variant (7 patients with c.1690C>T, 39 patients with c.1972C>T and 19 men with c.1977A>G) and of *BARD1* variant-negative prostate cancer patients (5650 non-carriers).

Category	Patients with c.1690 C>T Truncating Mutation (7 Cases)	Patients with c.1972 C>T Missense Variant (39 Cases)	Patients with c.1977 A>G Synonymous Variant (19 Cases)	BARD1 * Variant Negative Patients (5650 Cases)
Proportion of deceased	42.9% (3/7)	43.6% (17/39)	36.8% (7/19)	42.4% (2397/5650)
Median survival	nd	111	nd	140
5-year survival	71%	83%	73%	72%
10-year survival	57%	55%	49%	54%
Crude HR	0.95	0.94	0.92	
95%CI	0.31–2.92	0.59–1.51	0.44–1.92	ref.
*p*-value	0.93	0.81	0.82	
Age adjusted				
HR	0.96	0.87	1.06	
95% CI	0.21–2.96	0.54–1.40	0.51–2.22	ref.
*p*–value	0.94	0.56	0.87	

*—Reference group for OR and *p*-value calculations.

## Data Availability

The main research data supporting the results of this study are included in Table 1 Table 2 Table 3 and Figure 1; Figure 2. Other data can be made available upon reasonable request from the corresponding author.
